# Platelet Recruitment Promotes Keratocyte Repopulation following Corneal Epithelial Abrasion in the Mouse

**DOI:** 10.1371/journal.pone.0118950

**Published:** 2015-03-16

**Authors:** Fong W. Lam, Jenny Phillips, Paul Landry, Sri Magadi, C. Wayne Smith, Rolando E. Rumbaut, Alan R. Burns

**Affiliations:** 1 Baylor College of Medicine Department of Pediatrics, Section of Leukocyte Biology, 1100 Bates, CNRC 6058, Houston, Texas, 77030, United States of America; 2 Center for Translational Research on Inflammatory Diseases, Michael E. DeBakey VA Medical Center, 2002 Holcombe Blvd., MS 151, Houston, Texas, 77030, United States of America; 3 University of Houston College of Optometry, 505 J Davis Armistead Bldg, Houston, Texas, 77204, United States of America; 4 University of Wisconsin, Department of Pathology, 1300 University Ave., Room 6550, Madison, Wisconsin, 53706-1532, United States of America; The Chinese University of Hong Kong, HONG KONG

## Abstract

Corneal abrasion not only damages the epithelium but also induces stromal keratocyte death at the site of injury. While a coordinated cascade of inflammatory cell recruitment facilitates epithelial restoration, it is unclear if this cascade is necessary for keratocyte recovery. Since platelet and neutrophil (PMN) recruitment after corneal abrasion is beneficial to epithelial wound healing, we wanted to determine if these cells play a role in regulating keratocyte repopulation after epithelial abrasion. A 2 mm diameter central epithelial region was removed from the corneas of C57BL/6 wildtype (WT), P-selectin deficient (P-sel^-/-^), and CD18 hypomorphic (CD18^hypo^) mice using the Algerbrush II. Corneas were studied at 6h intervals out to 48h post-injury to evaluate platelet and PMN cell numbers; additional corneas were studied at 1, 4, 14, and 28 days post injury to evaluate keratocyte numbers. In WT mice, epithelial abrasion induced a loss of anterior central keratocytes and keratocyte recovery was rapid and incomplete, reaching ~70% of uninjured baseline values by 4 days post-injury but no further improvement at 28 days post-injury. Consistent with a beneficial role for platelets and PMNs in wound healing, keratocyte recovery was significantly depressed at 4 days post-injury (~30% of uninjured baseline) in P-sel^-/-^ mice, which are known to have impaired platelet and PMN recruitment after corneal abrasion. Passive transfer of platelets from WT, but not P-sel^-/-^, into P-sel^-/-^ mice prior to injury restored anterior central keratocyte numbers at 4 days post-injury to P-sel^-/-^ uninjured baseline levels. While PMN infiltration in injured CD18^hypo^ mice was similar to injured WT mice, platelet recruitment was markedly decreased and anterior central keratocyte recovery was significantly reduced (~50% of baseline) at 4–28 days post-injury. Collectively, the data suggest platelets and platelet P-selectin are critical for efficient keratocyte recovery after corneal epithelial abrasion.

## Introduction

The cornea is an avascular, optically transparent tissue that functions to filter incoming light and protect the rest of the eye from infection and blinding injuries. However, the cornea is highly susceptible to physical injuries, such as those occurring from epithelial abrasion or refractive surgeries. These injuries directly damage the stratified epithelial layer and cause keratocytes to die in the anterior region of the stroma beneath the affected site. While numerous studies have contributed to our current understanding of epithelial healing and keratocyte death [1–4], our understanding of keratocyte recovery is more limited. Keratocytes are resident stromal cells that actively synthesize essential extracellular matrix components including collagen, proteoglycans, and corneal crystallins, in order to sustain corneal clarity and preserve corneal ultrastructure [5,6]. There is ample evidence that keratocyte recovery is incomplete after corneal injury or surgery and their numbers do not reach normal levels for many years [1,2,7,8]. Incomplete recovery of keratocytes can lead to pathologic changes in the cornea; in fact, several studies report a high risk for corneal ectasia (thinning) in patients after LASIK (Laser-Assisted In situ Keratomileusis) which is associated with unusually low anterior keratocyte densities [1,8–11]. A better understanding of keratocyte recovery is relevant and important for preserving vision and may lead to strategies for improving keratocyte recovery after corneal epithelial injury.

In addition to epithelial cell and keratocyte death, corneal injury elicits an inflammatory response. After a simple, non-penetrating epithelial abrasion, an inflammatory cascade ensues whereby first-responding inflammatory cells, such as neutrophils (PMNs) and platelets, accumulate at the limbus; platelets remain at the limbus while PMNs enter the corneal stroma [3,12,13]. Whereas chronic inflammation can severely damage the corneal ultrastructure [9–11], an acute inflammatory response is beneficial and necessary for efficient corneal epithelial healing [3,12]. PMN extravasation is mediated by the leukocyte-specific β2 integrin, CD18; the complete absence of which delays PMN infiltration and epithelial wound closure [4,12,14]. The adhesion molecule P-selectin (CD62P) plays an important role in PMN and platelet recruitment [3,15,16] in the injured cornea. The absence of P-selectin significantly impairs platelet accumulation at the limbus and this is associated with delayed epithelial wound closure [3,16]. PMN and platelet accumulation at the limbus are coincident and co-dependent responses. Antibody-induced selective depletion of circulating platelets or PMNs results in a significant reduction in recruitment of the other cell type and delays corneal epithelial healing, demonstrating that platelets, PMNs, or both, are needed for epithelial recovery [3]. Collectively, these observations support the concept that inflammatory cell recruitment is important in corneal epithelial healing.

Since platelets and PMNs are critical for corneal epithelial wound healing, we wished to determine if they also participate in keratocyte recovery following a central corneal epithelial abrasion.

## Material and Methods

### Animals

Male C57/BL6 wildtype (WT) mice, P-selectin^-/-^ mice (P-sel^-/-^; B6.129S7-Selp^*tm1Bay*^), and CD18 hypomorphic mutant mice (referred to as CD18^hypo^; B6.129S7-*Itgb2* ^*tm1Bay*^) were purchased from The Jackson Laboratories (Bar Harbor, ME). All mice were housed and bred at the Baylor College of Medicine animal housing facilities. The CD18^hypo^ mice were originally developed at Baylor College of Medicine by Arthur Beaudet’s laboratory and have been backcrossed at least 10 generations with C57BL/6J mice [17]. As opposed to complete knock-out mice (such as B6.129S7-*Itgb2* ^*tm2Bay*^), resting granulocytes from CD18^hypo^ mice express CD18, albeit at levels <2% of WT baseline CD18 expression. After phorbol 12-myristate 13-acetate (PMA) activation, CD18 expression on CD18^hypo^ granulocytes remains low (16% of WT) [17]. CD18^hypo^ mice are less susceptible to spontaneous bacterial infections compared to CD18 null mice, making them more suitable to study the role of CD18 in inflammatory disease processes [18,19].

### Ethics statement

For wound healing studies, 3 to 10 male mice per genotype and per study, ages 6 to 12 weeks, were used. All animals were treated according to the guidelines described in the ARVO Statement on the Use of Animals in Ophthalmic and Vision Research and were reviewed and approved by the Baylor College of Medicine Institutional Animal Care and Use Committee policy (Protocol Number: AN-2721) sand Animal Care and Use Policies of the University of Houston (Protocol Number: 13-003). All surgery was performed under pentobarbital anesthesia, and all efforts were made to minimize suffering. Euthanasia was performed using pentobarbital overdose followed by cervical dislocation.

### Wound protocol

Mice were anesthetized by administering pentobarbital (50 mg/kg body weight) by intraperitoneal (i.p.) injection. The central corneal epithelium was abraded as previously described [16,20]. Briefly, a 2 mm diameter trephine was used to demarcate the central epithelial region of the right eye and the epithelium within the demarcated region was mechanically removed using an Algerbrush II (Alger Equipment Co., Inc., Lago Vista, TX) while viewing the cornea under a dissecting microscope. The corneal epithelial abrasion injury model has been used for over a decade to evaluate corneal inflammation, epithelial healing, stromal recovery, and keratocyte death and recovery [4,12,14,21–23]. Uninjured right corneas were obtained from separate sets of mice of each genotype for all baseline measurements.

### Passive transfer of platelets

For each recipient mouse, blood pooled from 3–4 donor mice (WT or P-sel^-/-^) was used to isolate platelets following an established protocol [3,16,24]. Briefly, under isoflurane anesthesia, approximately 0.9 mL of blood was collected into a syringe containing 0.1 mL acid-citrate-dextrose (ACD; Sigma-Aldrich, St. Louis, MO) for anticoagulation and transferred into polypropylene tubes. The blood was centrifuged at 260*g* for 8 minutes to collect platelet-rich plasma (PRP; supernatant). Platelets were then pelleted from PRP by centrifugation at 740*g* for 10 minutes. The platelet pellets were gently resuspended in 500 μL of PBS and allowed to rest at room temperature for up to 30 minutes, during which platelets were counted using a hemocytometer. The platelet concentration was adjusted to 10^6^ platelets/μL in 200 μL aliquots and injected into the tail vein of recipient mice (WT or P-sel^-/-^) immediately before corneal epithelial abrasion. This method of platelet isolation and been shown by others [25] and us [3,24] to result in less than ~0.01% leukocytes in the platelet suspension.

### Immunofluorescence staining

**Corneal immunostaining for keratocyte counting.** Following corneal abrasion, mice were euthanized with a lethal dose of pentobarbital (120 mg/kg i.p.) followed by cervical disarticulation at the following time points: 1, 4, 14, and 28 days after injury. Uninjured and injured right corneas from these mice were excised and fixed in 2% paraformaldehyde (Tousimus Research Corporation, Rockville, MD) in phosphate buffered saline (PBS, pH 7.2) for 60 minutes at 4° C, blocked for 30 minutes in PBS with 2% BSA, and permeabilized for 30 minutes with 0.1% Triton-X. Corneas were then incubated with a cocktail of leukocyte-specific antibodies (each conjugated with FITC) against CD3e, CD4, CD8, CD18, CD11a, CD11b, CD45, F4/80, GR1, Ly6G, and γδ TCR (BD Bioscience). Cell nuclei were stained with 4',6-diamidino-2-phenylindole (DAPI; Sigma, St. Louis, MO). This approach was necessary because specific, reliable markers for mouse keratocytes are lacking. The two most reliable keratocyte-specific markers are keratocan, a corneal-specific proteoglycan synthesized and secreted by keratocytes, and aldehyde dehydrogenase (ALDH) 3A1, a corneal crystallin also synthesized by keratocytes [5,6]. Since keratocan is secreted throughout the stroma, keratocan immunostaining results in intense stromal staining making it difficult to visualize individual keratocytes and obtain reliable cell counts. Although ALDH3A1 staining localizes to the keratocyte cell body [20], cell to cell staining is inconsistent. In the present study, since keratocytes and leukocytes comprise the vast majority of nucleated cells within the inflamed corneal stroma, nucleated cells that did not stain with the FITC-cocktail of leukocyte-specific antibodies were considered to be keratocytes.

**Preparation of corneas for analysis of PMN and platelet recruitment.** Following euthanasia, right corneas from uninjured and injured mice at 6h intervals through 48h (*n* = 4 to 6 corneas per time point, for each genotype) were excised and prepared for immunofluorescence microscopy as described above. All corneas were then incubated overnight at 4° C with the following fluorescently-labeled antibodies for platelets, PMNs, blood vessels, and nuclei, respectively: anti-CD41/PE (GP IIb; BD Bioscience, San Jose, CA), anti-Ly6G/FITC (BD Bioscience, Pharmingen, San Jose, CA), anti-CD31/APC (PECAM-1; BD Bioscience, Pharmingen, San Jose, CA), and DAPI.

**Corneal imaging.** Following overnight antibody incubations, all corneas were washed three times in PBS and radial cuts were made from the peripheral edge toward the paracentral region. Corneas were flat mounted in AIRVOL (Celanese, Dallas, TX) or Prolong Gold self-curing mounting media (Invitrogen, Carlsbad, CA), and imaged with a DeltaVision Spectris Core inverted fluorescence microscope (Applied Precision, Issaquah, WA).

### Morphometry

**Fig 1:**
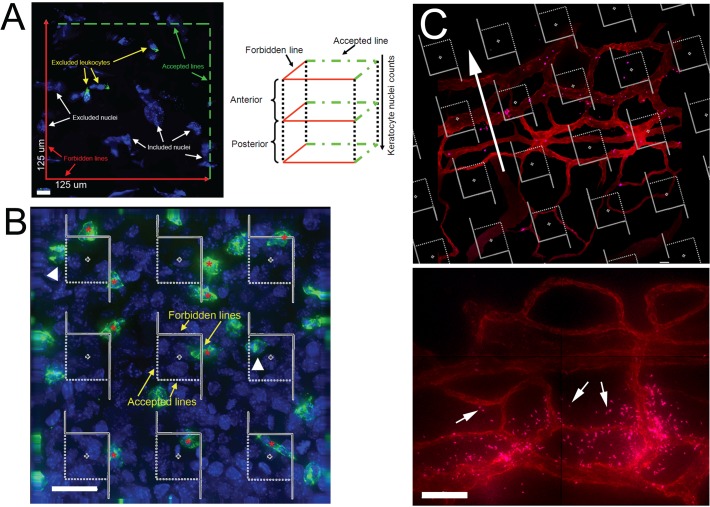
Morphometric analysis of keratocytes, PMNs, and platelets. (A) A morphometric number estimation box to count keratocyte nuclei through the thickness of the stroma. Non-FITC stained cells with blue fluorescing nuclei were considered keratocytes and were counted if the entire nucleus was contained within the box or on the accepted lines (dashed green lines; top and right). Nuclei that contacted the forbidden lines (solid red lines; bottom and left) were excluded. Scale bar = 10 μm. (B) A 3 x 3 morphometric number estimation grid used to determine PMN density (PMN/mm^2^). Each grid square had an area of 625 μm^2^. All PMN nuclei within the grid box or on the accepted line were counted (white arrowheads) whereas PMN nuclei outside the grid boxes or on the forbidden line were not counted (red asterisk). Scale bar = 25 μm. (C) Upper panel: a 10 x 6 number estimation grid was oriented perpendicular to the major axis (arrow, top panel) of the limbal vessels. Lower panel: extravascular platelets (arrows) were identified and enumerated to determine platelet density. Scale bar = 50 μm.

**Morphometric analysis of keratocyte recovery.** To minimize observer bias, cell counts were obtained through the full thickness of the corneal stroma using a systematic uniform random sampling (SURS) strategy, as illustrated in Fig. 1A. This approach is robust and considered to be a well-validated method for unbiased enumeration [26–29]. For keratocyte counts, a morphometric number estimation box (125 x 125 μm; 1.56 x 10^4^ μm^2^) was used to count only non-FITC-labeled (DAPI-stained) cell nuclei falling within the frame of the estimation box or on the accepted lines, through the thickness of the stroma. All non-FITC-labeled (DAPI-stained) nuclei falling on the forbidden lines were excluded from the counts. Central and paralimbal keratocyte numbers were analyzed in uninjured and 1, 4, 14, and 28 day post-injured corneas.

**Morphometric analysis of PMN recruitment.** Images were obtained through the full thickness of four limbal and paralimbal regions and a single central corneal region. A morphometric number estimation 9-square grid (25 x 25 μm squares, 625 μm^2^/square) was overlaid upon the single panel 2.72 x 10^4^ μm^2^ image (Fig. 1B). Only Ly6G/FITC-positive PMN nuclei falling within the grid squares or on the accepted frame lines, but not on forbidden frame lines, were counted through the thickness of the image stack. PMN density, expressed in PMNs/mm^2^, was defined as the total number of counted PMNs divided by the total sampling area, converted to mm^2^.

**Morphometric analysis of platelet recruitment.** A 9-panel image was acquired through the thickness of the limbal vessels at 40X magnification using the DeltaVision Spectris Core fluorescence microscope. The images were post-processed into 2.25 X 10^5^ μm^2^ montages using Softworx software (Applied Precision, Issaquah, WA). Digital images were analyzed in Adobe Photoshop (Adobe Systems Inc., San Jose, CA) after randomly casting a 60-square (10 X 6; 3,211 μm^2^/square) morphometric number estimation grid (Fig. 1C) over the montage. The grid squares were oriented perpendicularly to the major axis of the limbal vessels. As platelets enter the extravascular space they degranulate and fragment as they interact with the matrix. For analyses, only CD41-positive extravascular platelets greater than 2 μm in diameter were counted as these represent intact whole platelets that have yet to fragment. Within platelet aggregates, a 2-μm diameter region was considered an individual platelet and total platelet number within an aggregate was estimated as the sum of 2-μm areas within an aggregate. Only platelets falling within the grid boxes or on the accepted lines of the grid boxes were counted. Platelet density, expressed in platelets/mm^2^, was defined as the total number of platelets counted divided by the total sampling area, converted to mm^2^.

### Statistical analysis

Data were analyzed using one-way or two-way analysis of variance (ANOVA) with Bonferroni’s multiple comparison post-test, as appropriate, using Prism software (GraphPad Software, Inc.). A *p* value ≤0.05 was considered statistically significant; data are reported as means ± SEM.

## Results

### Keratocyte recovery in wildtype mice after corneal epithelial injury is incomplete

**Fig 2:**
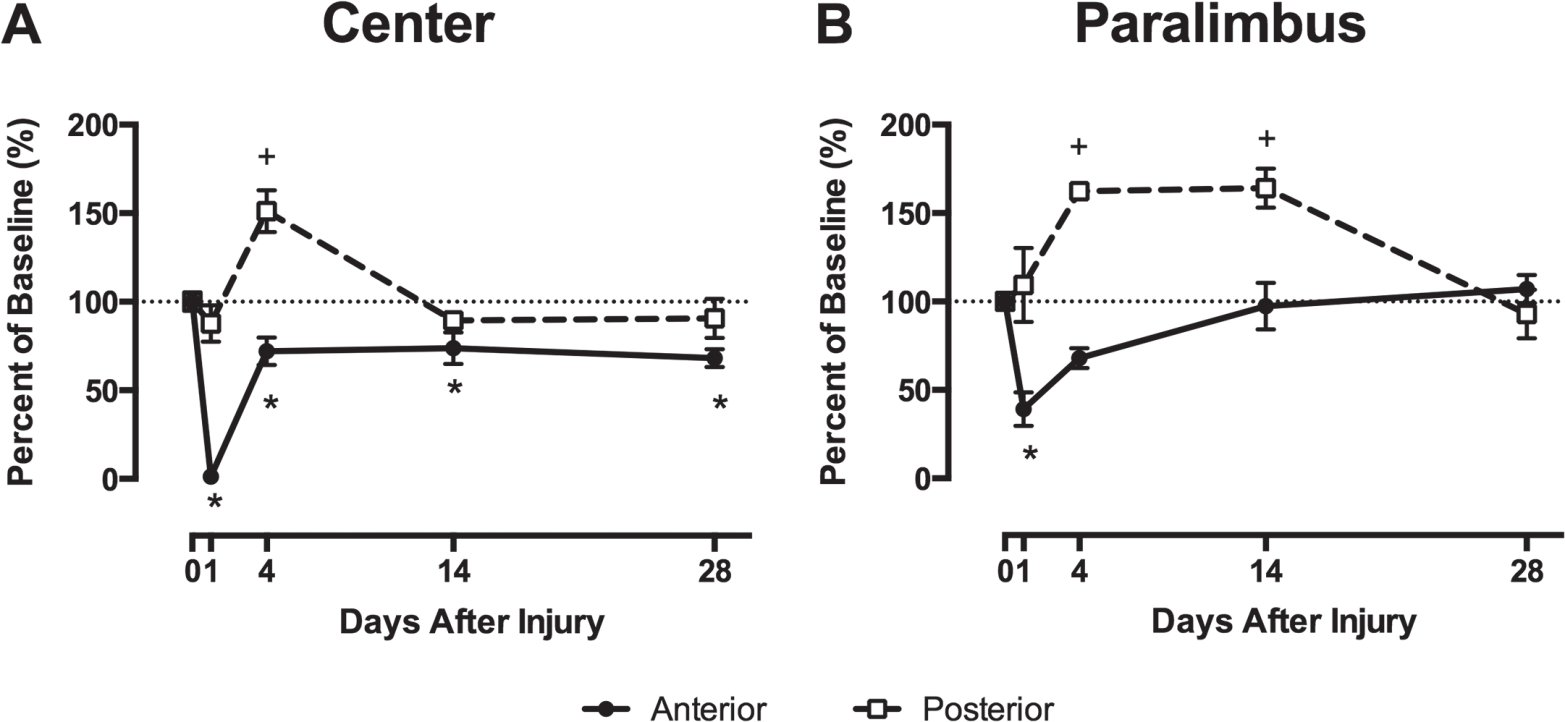
Anterior central keratocyte recovery is incomplete after corneal epithelial abrasion. (A) Anterior keratocyte counts in the central region (solid line) dropped and remained below baseline values out to 28 days post-injury (*p≤0.05), whereas posterior central keratocyte counts (dashed line) increased after injury at 4 days (^+^p≤0.05) before returning to baseline levels. (B) Anterior paralimbal keratocytes (solid line) decreased initially (*p≤0.05), but returned to baseline by 28 days. Posterior paralimbal keratocytes (dashed line) were elevated by 4 and 14 days (^+^p≤0.05), but returned to baseline by 28 days. Results are mean percentages of baseline keratocyte counts ± S.E.M. N = 5 mice per time point.

In this first set of experiments, we were interested in characterizing the keratocyte recovery response after epithelial abrasion in the mouse cornea. For ease of interpretation, keratocyte counts are expressed as a percentage of the baseline counts found in uninjured corneas; baseline keratocyte counts prior to injury are provided in Table 1. At the injury site, anterior keratocytes in WT mice were absent at one day after injury, as expected. By four days, anterior central keratocyte counts recovered to 72 ± 8% of baseline and maintained that level through 28 days (68 ± 5% of baseline); baseline levels were never fully restored during this period (Fig. 2A). Posterior central keratocytes in WT mice also responded to the injury by changing their cell numbers, increasing to 151% ± 12% of baseline four days after injury. The increase in keratocyte counts was transient, and by 28 days, posterior central keratocyte counts returned to baseline levels.

**Table 1:** Baseline keratocyte counts prior to injury.

Genotype	Center		Paralimbus	
	Anterior	Posterior	Anterior	Posterior
Wildtype	46.6 ± 1.5	36 ± 1.9	42.9 ± 2.1	33.7 ± 1.2
P-sel^-/-^	44.3 ± 2.1	37.3 ± 4.5	41.2 ± 0.6	33.5 ± 2.4
CD18^hypo^	47.4 ± 3.9	45.6 ± 3.4	51.1 ± 4.6	47.2 ± 3.9*

Numbers are mean counts ± SEM.

*P≤0.05 vs. posterior paralimbus keratocyte counts in wildtype and P-sel^-/-^ mice using 2-way ANOVA with Bonferroni’s multiple comparison test, n = 3–5 mice per group.

In contrast to the central region, the keratocyte response in the paralimbal region where the epithelium was not removed by the Algerbrush was quite different. In the anterior paralimbal region, keratocyte counts fell to 39% ± 9.5% of baseline one day after injury. While the rate of recovery was slower than in the anterior central region, there was a return toward baseline on day 4 post-injury and complete recovery to baseline by 14 days which was maintained to 28 days post-injury (Fig. 2B). In the posterior paralimbal region, the keratocyte count increased 4 days after injury, and remained elevated at 14 days (164 ± 11% of baseline). By 28 days, posterior paralimbal keratocyte counts returned to baseline (Fig. 2B).

### Platelets and platelet P-selectin are important for keratocyte recovery

**Fig 3:**
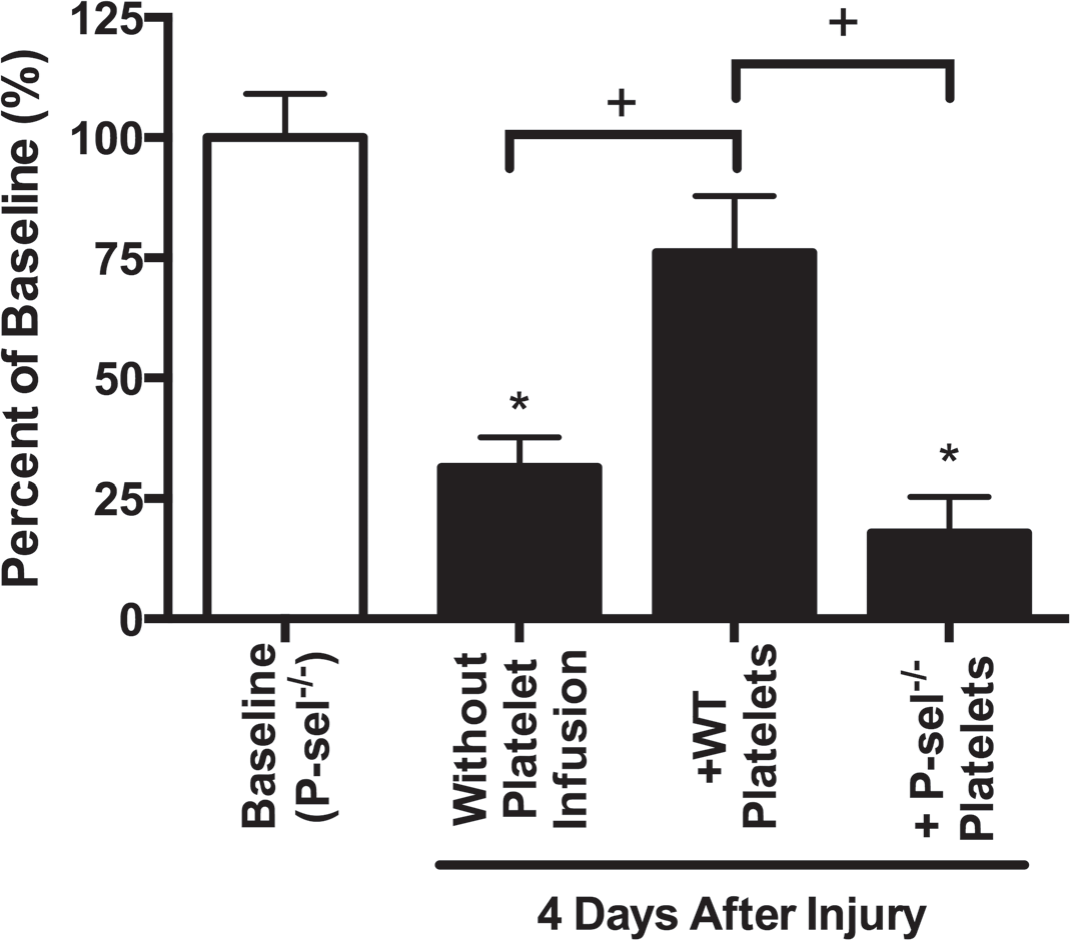
Platelet P-selectin is necessary for keratocyte recovery after corneal epithelial abrasion. Anterior central keratocyte counts (expressed as percent of baseline) in P-sel^-/-^ mice at 4 days post-injury were decreased (*p≤0.05). Infusion of WT platelets prior to injury increased keratocyte recovery at 4 days post-injury compared to no infusion (^+^p≤0.05). In contrast, P-sel^-/-^ mice receiving P-sel^-/-^ platelets had significantly reduced keratocyte recovery compared to baseline (*p≤0.05), similar to mice receiving no infusion, and this was significantly lower than keratocyte recovery in mice receiving WT platelets (^+^p≤0.05). N = 3–10 mice per group.

P-sel^-/-^ mice have decreased PMN and platelet recruitment which is accompanied by diminished epithelial wound healing after corneal abrasion [3,16]. Four days after corneal abrasion, anterior central keratocyte recovery in P-sel^-/-^ mice was markedly reduced compared to WT mice (31 ± 6% vs. 72 ± 8%, p ≤0.05). Delayed epithelial wound healing in P-sel^-/-^ mice can be reversed by passive transfer of WT platelets bearing P-selectin[3]. As shown in Fig. 3, passive transfer of WT platelets improved keratocyte recovery in P-sel^-/-^ mice. To eliminate the possibility that platelets were potentially activated during the isolation and transfusion processes, we performed control experiments with passive transfer of platelets derived from P-sel^-/-^ mice. Transfusion with P-sel^-/-^ platelets into P-sel^-/-^ mice had no effect on keratocyte recovery (Fig. 3). Collectively, these studies show platelets and the platelet-associated adhesion molecule P-selectin are clearly linked to keratocyte recovery.

### PMNs alone are not sufficient for keratocyte recovery after injury in CD18^hypo^ mice

**Fig 4:**
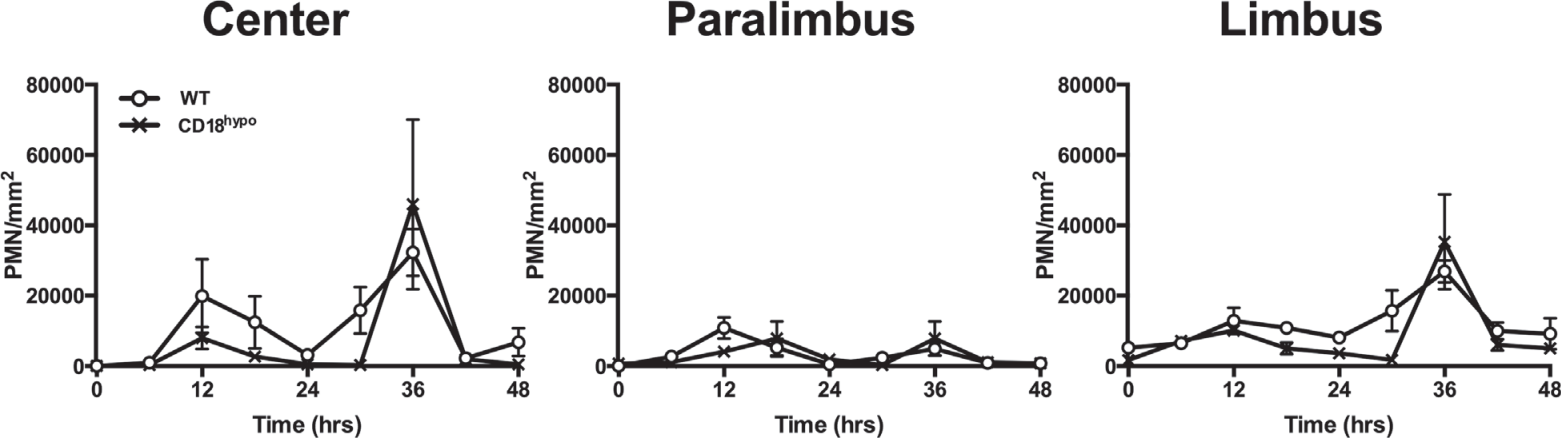
PMN recruitment is normal in mice expressing low levels of CD18. The recruitment of PMNs is similar between CD18^hypo^ (x) and WT (o) mice in the center, paralimbus, and limbus regions of the cornea from 0 to 48h after injury. N = 3–6 mice per genotype per time point.

**Fig 5:**
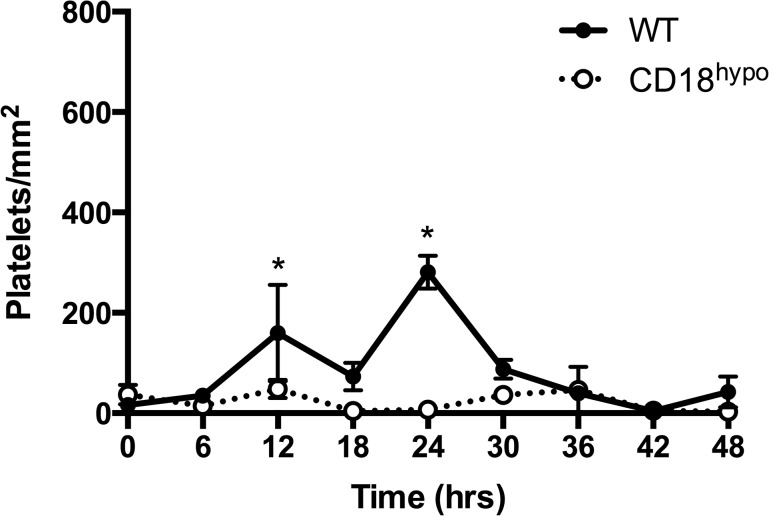
CD18 is necessary for platelet recruitment to the injured cornea. Platelet counts were low and similar between uninjured corneas from CD18^hypo^ and WT mice. In WT mice at 12 and 24h post-injury, there was a significant increase in recruited platelets at the limbus (*p≤0.05), but not in CD18^hypo^ mice. N = 3–5 mice per genotype per time point.

**Fig 6:**
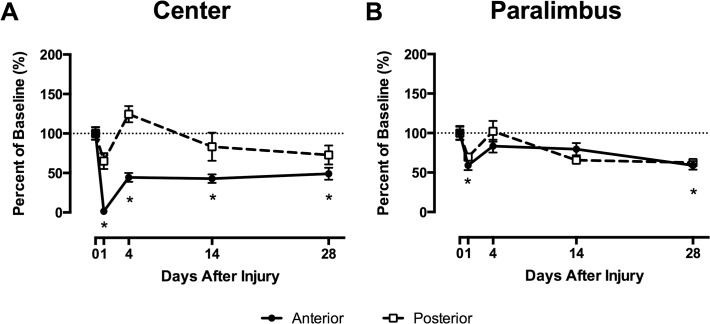
CD18^hypo^ mice have impaired keratocyte recovery after corneal epithelial abrasion. (A) Anterior central keratocyte recovery (solid line) in CD18^hypo^ mice is only 50% of baseline values by 28 days post-injury, whereas the posterior (dashed line) region was not significantly different from baseline. (B) In the paralimbal region, where there was no direct epithelial injury, anterior keratocyte counts decreased by 40% and were not recovered at 28 days post-injury. *p≤0.05 compared to uninjured levels. N = 3–5 per time point.

Although the experimental outcomes described above are consistent with a role for platelets in keratocyte recovery, impaired platelet recruitment is associated with decreased PMN recruitment [3,4,16]. Hence, the observed decrease in keratocyte recovery in P-sel^-/-^ mice may be attributable to diminished PMN recruitment, which is also restored in P-sel^-/-^ mice following passive transfusion with WT platelets [3]. To determine if PMNs alone make a significant contribution to keratocyte recovery, we chose to study hypomorphic CD18 (CD18^hypo^) mice. Previously, we reported that mice with a complete deficiency in CD18 (CD18^-/-^) had decreased PMN infiltration [12,14]. In contrast, the small amount of CD18 expressed on PMNs in CD18^hypo^ mice was sufficient for PMN infiltration after corneal abrasion and the level of infiltration after wounding was not different from that in WT mice (Fig. 4). In WT mice, platelet recruitment at the limbus peaks at 12 and 24h post-corneal abrasion [3,4,16]. As expected, Fig. 5 shows the limbal region of WT mice had significant platelet accumulation at 12 and 24h post-injury. However, platelet accumulation at the limbus was markedly reduced in injured CD18^hypo^ mice and never increased above baseline levels through the 48h observation period (S1 Fig.). WT and CD18^hypo^ mice had similar circulating PMN (0.52 ± 0.03 vs 1.03 ± 0.31 x10^3^/μl, respectively, p = 0.171) and platelet counts (1,153 ± 211 vs. 1,334 ± 48 x10^3^/μl, respectively, p = 0.448). Importantly, in this setting where PMN accumulation was normal and platelet accumulation was low, central anterior keratocyte recovery in CD18^hypo^ mice was significantly less compared to WT mice at 4 days post-injury (44 ± 6% vs. 72 ± 8%, respectively, p≤0.05) and keratocyte recovery failed to improve by 28 days post injury (Fig. 6). Moreover, posterior central keratocyte counts in CD18^hypo^ mice did not increase significantly after injury as they did in WT mice (compare Fig. 6 with Fig. 2). Finally, anterior and posterior keratocyte numbers in the paralimbal region were significantly below baseline levels at 28 days post-injury (Fig. 6). Collectively, these data suggest platelets are necessary for keratocyte recovery after corneal abrasion.

## Discussion

Corneal injury is clearly a high risk factor to vision loss and of major concern to clinicians. Whereas epithelial healing has been extensively studied by our lab and others [3,12,13,22,23,30,31], keratocyte recovery following corneal injury is not as well understood. The purpose of this study was to determine if recruited inflammatory cells participate in keratocyte recovery following a central corneal epithelial abrasion. Specifically, since PMN and platelet recruitment to the injured cornea is critical for efficient epithelial wound healing, we wanted to know if these inflammatory cells also play a role in keratocyte repopulation. Collectively, the experiments outlined in the current study identify a role for platelets rather than PMNs in supporting efficient keratocyte recovery. This is clearly supported by three lines of evidence: 1) in P-sel^-/-^ mice, where platelet and PMN recruitment are diminished, anterior central keratocyte recovery is depressed compared to WT mice; 2) in P-sel-/- mice, after passive transfer of WT platelets, the diminished keratocyte response is restored to WT levels, and; 3) in CD18^hypo^ mice, where PMN recruitment is normal but platelet recruitment is markedly reduced, anterior central keratocyte recovery is depressed compared to WT mice. To our knowledge, this is the first study to demonstrate a specific role for platelets in keratocyte repopulation after corneal injury.

These findings are consistent with previous corneal healing studies in P-sel^-/-^ mice which also demonstrated that platelet recruitment during the first 24h after corneal abrasion plays an important role in corneal epithelial cell division and wound closure [3]. That study also showed platelet P-selectin, and not endothelial P-selectin, was linked to diminished epithelial wound healing and this agrees with the current passive transfer experiments of WT platelets into P-sel^-/-^ mice where keratocyte recovery is restored to uninjured baseline levels. Platelet P-selectin appears to be critical for platelet accumulation at the limbus after corneal abrasion. Platelets adhere to PMNs and enhance PMN migration via platelet P-selectin binding to P-selectin glycoprotein ligand-1 (PSGL-1) expressed on the PMN [16]. While the current study suggests an essential role for platelets rather than PMNs in keratocyte recovery, PMN recruitment in WT mice appears to be necessary for platelet recruitment [3].

In the current study, we evaluated long-term (28 day) keratocyte recovery in two distinct regions of the cornea: at the site of injury (center) as well as at a site not directly injured by abrasion (paralimbus). The number of keratocytes in the center was lowest 24h after epithelial abrasion but increased thereafter. This is consistent with data reported by Zieske *et al*. demonstrating an increase in keratocyte proliferation starting 24h after injury at the wound edge and in the center [21]. In that study, peak keratocyte proliferation occurred at 44h after injury, after which point it declined, with cell division not evident at 7 days post-injury. This agrees with our current observation that the number of keratocytes in the wounded area does not increase after 4 days nor does it return to baseline, even after 28 days. Interestingly, we also observed an initial decrease in anterior paralimbal keratocyte counts, despite the fact that the region is remote from the site of injury. One interpretation is that peripheral keratocytes begin their proliferation and migration early as they move toward the central wound to replace lost keratocytes.

In humans, loss of anterior keratocytes is evident after extensive contact lens wear and refractive surgery with incomplete keratocyte repopulation [1,7,32,33]. While the source of the repopulating cells is unclear, several possibilities exist, including peripheral keratocytes, resident limbal stem cells, or bone marrow-derived blood borne mesenchymal stem cells which may infiltrate the injured cornea [34]. Regardless of the source of repopulating keratocytes, there is ample evidence from the literature supporting the concept that platelet-derived growth factors play a role in keratocyte expansion. Growth factors are essential for many aspects of wound healing including cell proliferation, migration and/or cellular differentiation [35–40] and platelets are an excellent source of growth factors [41,42]. In our corneal abrasion model, platelet counts rise and fall over the 48h post-injury period. The fall in platelet counts likely reflects platelet activation upon contact with the extracellular matrix, which would lead to platelet degranulation and fragmentation. Degranulation would release growth factors into the limbus and peripheral cornea, which contains keratocytes that have not been directly injured by the central epithelial abrasion. Transforming growth factor (TGF)-β and platelet-derived growth factor (PDGF) are found in platelet α granules and have been shown to promote keratocyte differentiation *in vitro* [37–40,43]. As well, Etheredge and colleagues demonstrated that addition of insulin-like growth factor-1 (IGF-1), TGF-β or PDGF, three growth factors known to be present in platelet granules, enhanced keratocyte DNA synthesis *in vitro* [44]. *In vivo*, topical application of platelet extracts not only promotes epithelial recovery but it also triggers differentiation of keratocytes into myofibroblasts [36,45]. In the present study, keratocyte transformation into the myofibroblast phenotype was not observed (repopulating keratocytes were negative for smooth muscle α-actin; data not shown). Why this occurs with topical applications is unclear, but it may relate to the site and method of application. Endogenous platelet-secreted growth factors would naturally localize to the limbus, the site of extravasation, whereas topically applied growth factors would have access to the entire cornea. Additionally, the presence of platelets themselves, and not just their growth factors, may play an important role in promoting keratocyte repopulation without myofibroblast transformation. This is suggested by a recent study in which topically applied platelet rich plasma appeared to decrease the number of myofibroblasts after PRK as compared to no treatment [46]. If platelet-derived growth factors are to be pursued as potential therapeutic agents for promoting keratocyte recovery, it may be important to consider whether it is possible to target their delivery to the limbus (e.g., it induces keratocyte transformation into fibroblasts and this compromises corneal transparency).

In summary, our data suggest that platelets and platelet P-selectin are critical for efficient keratocyte recovery after corneal epithelial abrasion.

## Supporting Information

S1 Fig Fluorescence image of the limbal region in WT (Panels A-C) and CD18^hypo^ mice (Panels D-F) 24 hours after corneal epithelial abrasion injury. WT mice had more platelet recruitment (arrows; magenta) in the limbus (vessels stained in red) as compared to CD18^hypo^ mice despite similar number of neutrophils (green). Scale bar = 40 μm.
(TIF)
