# Supplementary figures and images for: Platelet Recruitment Promotes Keratocyte Repopulation following Corneal Epithelial Abrasion in the Mouse
Source: PLoS One. 2015 Mar 16;10(3):e0118950. doi: 10.1371/journal.pone.0118950 (PMC4361664; doi:10.1371/journal.pone.0118950)

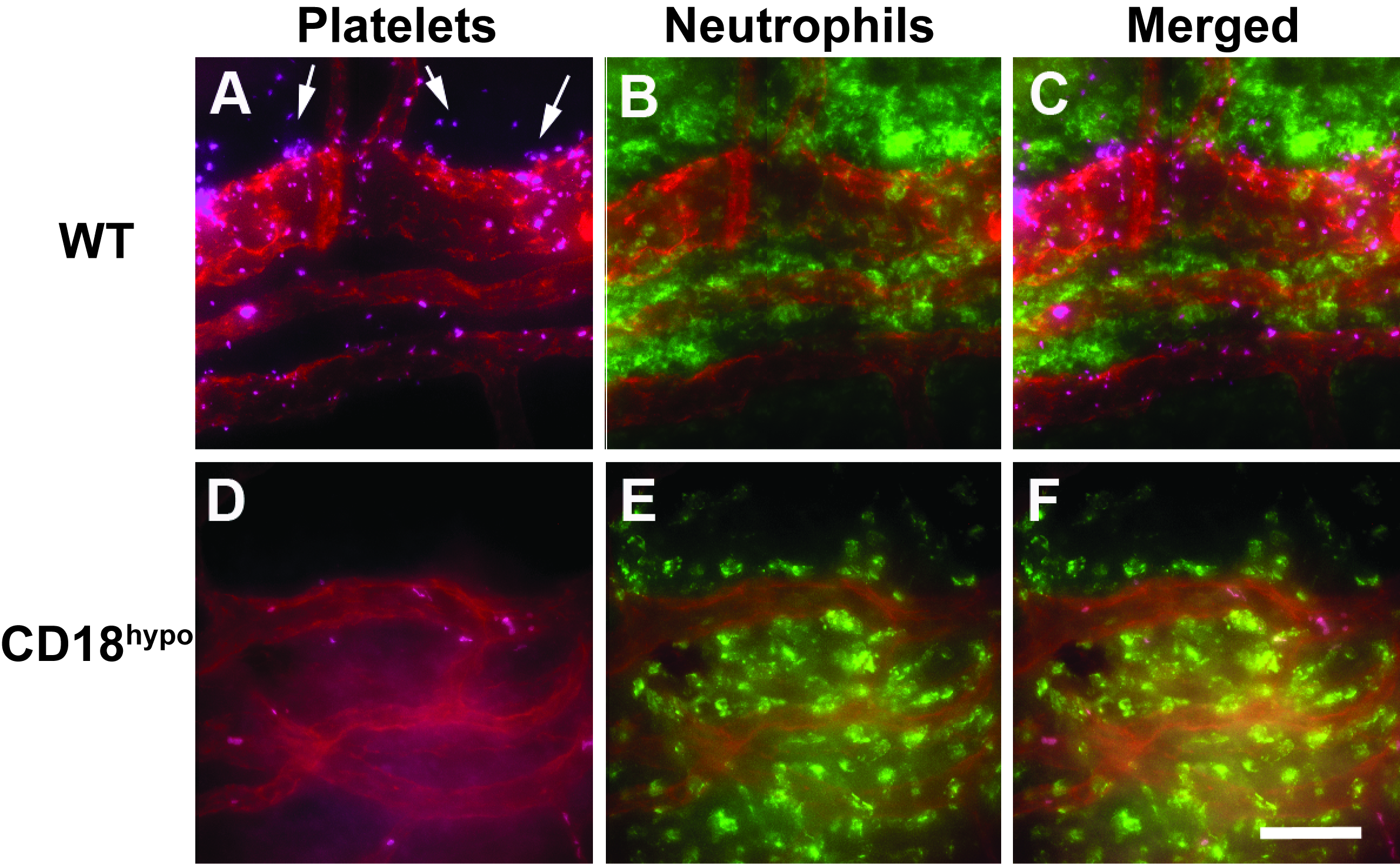

Supplement: S1 Fig — WT mice had more platelet recruitment (arrows; magenta) in the limbus (vessels stained in red) as compared to CD18hypo mice despite similar number of neutrophils (green). Scale bar = 40 μm. (TIF) [file pone.0118950.s001.tif]
